# Adverse Drug Reactions in Hospitalized Patients: Results of the FORWARD (Facilitation of Reporting in Hospital Ward) Study

**DOI:** 10.3389/fphar.2018.00350

**Published:** 2018-04-11

**Authors:** Claudia Giardina, Paola M. Cutroneo, Eleonora Mocciaro, Giuseppina T. Russo, Giuseppe Mandraffino, Giorgio Basile, Franco Rapisarda, Rosarita Ferrara, Edoardo Spina, Vincenzo Arcoraci

**Affiliations:** ^1^Department of Clinical and Experimental Medicine, University of Messina, Messina, Italy; ^2^Regional Pharmacovigilance Centre, University Hospital of Messina, Messina, Italy; ^3^Department of Pharmacy, Catania Local Health Service, Catania, Italy

**Keywords:** adverse drug reactions, pharmacovigilance, preventability, hospital admission, elderly, internal medicine, risk factors

## Abstract

**Background:** Adverse drug reactions (ADRs) are an important public health problem, representing a major cause of morbidity and mortality. However, several countries have no recent studies available. Since 2014, a prospective active pharmacovigilance project, aimed to improve ADRs monitoring in hospital wards (FORWARD) was performed in Sicily. This study, as part of FORWARD project, was aimed to describe ADRs occurred during the hospital stay in Internal Medicine wards. ADRs related to hospital admission, characteristics and preventability of ADRs were also evaluated.

**Methods:** Demographic, clinical, and pharmacological data on patients admitted to six wards of Internal Medicine, from 2014 to 2015, were collected by trained, qualified monitors, who screened all medical records. The rate of ADRs occurred during hospital stay and those leading to hospitalization were analyzed. A descriptive analysis of the reactions, suspected drugs, and associated factors was performed according to the setting analyzed.

**Results:** During the study period, 4,802 admissions were recorded; in 3.2% of them ADRs occurred during hospital stay while in 6.2%, admission was due to ADRs. The duration of hospital stay was longer in patients who experienced ADRs during hospitalization, compared to patients without ADRs [median days 12 (Q1–Q3: 8–17) vs. 9 (6–13)]; *p* < 0.001). Females [OR1.39 (95% CI 1.03–1.93)] and patients taking ≥ 4 drugs [OR1.46 (95% CI 1.06–2.03)] were more likely to experience ADRs during hospital stay, as well as to be admitted because of ADRs [female: OR1.75 (95% CI 1.37–2.24); ≥ 4 drugs: OR2.14 (95% CI 1.67–2.74)]. The most frequent ADRs occurred during hospital stay were *cutaneous* (26.8%), *general* (13.4%), *vascular* (13.4%), and *cardiac* (11.5%) disorders and the drug classes mainly involved were anti-bacterials (38.2%) and antithrombotic agents (21.7%). ADRs were serious in 44.6% and probably preventable in 69.4%. *Gastrointestinal* (27.7%), *hematological* (26.5%), *metabolic* (18.1%), and *nervous* (16.1%) disorders were the main ADRs cause of hospitalization, primarily due to antithrombotic agents (39.0%) RAS-inhibitors (13.9%), NSAIDs (11.9%), and diuretics (9.0%). Only 12.9% of them was not preventable.

**Conclusion:** Adverse drug reactions occurred during hospitalization or contributing to admission to Internal Medicine wards were considerable and most of them were preventable. Females and patients taking many medications were more likely to present ADRs both during hospital stay or as cause of admission.

## Introduction

An adverse drug reaction (ADR) is defined as a noxious, unintended injury that arise from drug related causes. The growing evidence on the increased frequency and severity of ADRs, associated with a negative impact on patient’s health status, also reveals that ADRs entail a significant burden on healthcare facilities, increasing the length of hospital stay, and requiring sometimes additional investigations and drug therapies for the treatment of symptoms and diseases caused to the patient (Classen et al., 1997; Pirmohamed et al., 2004; Aronson, 2013; Sultana et al., 2013; European-Medicines-Agency, 2014). ADRs account for 5–10% of all hospital admissions (Beijer and de Blaey, 2002; Baker et al., 2004; Kongkaew et al., 2008), depending on the individual study designs, the study population and the definition of ADR used (Kongkaew et al., 2008; Al Hamid et al., 2014). Furthermore, differences in available medicines and medical practice could result in different ADR frequencies detected from epidemiological studies in European and United States hospitals. On the basis of a survey ordered by European Commission, it has been estimated that 5% of hospital admissions in Europe are due to ADRs, 5% of all hospitalized patients experience an ADR during hospital stay and ADRs represent the fifth most common cause of death in hospital setting. Moreover, 197,000 deaths per year in the European Union are caused by ADRs and the total cost to society of ADRs in the EU is €79 billion (2008). A landmark meta-analysis conducted by Lazarou et al. (1998) found that ADRs were the fourth to six highest cause of death in the United States, following ischemic cardiopathy, cancer, and stroke. In relation to mortality, Davies et al. (2009) found an increased risk in patients who experienced an ADR compared to those who did not.

The characteristics of the population as gender or advanced age and the right use of drugs play a key role in the development of ADRs. Indeed, ADRs risk significantly increase in case of inappropriate prescriptions or drug–drug interactions (Piacentini et al., 2005; Trifiro et al., 2006, 2008a,b; Alacqua et al., 2008, 2009; Cavagna et al., 2013; Ferrajolo et al., 2014; Italiano et al., 2015; Rafaniello et al., 2015). Approximately 50% of ADRs are preventable (Davies et al., 2009; Pourseyed et al., 2009; Farcas et al., 2010; Hakkarainen et al., 2012), providing strong evidence that post-marketing drug surveillance plays an increasingly important and essential role, mainly in assessing benefit/risk ratios, health economics, and public health.

Despite the large number of data, no recent studies on the impact of ADRs on hospitalized patients, particularly in the internal medicine environment, are available. The few available studies have shown that iatrogenic events occur in 14–25% of hospitalized patients and in 33% of patients over 65 years of age (de la Sierra et al., 1989; Sampereiz Legarre et al., 1994; Gonzalez-Martin et al., 1997; Madeira et al., 2007; Mohebbi et al., 2010). Moreover, only few studies specifically evaluated the factors associated with the in-hospital adverse events occurrence (Fauchais et al., 2006; Madeira et al., 2007; Mohebbi et al., 2010; Dupouy et al., 2013). Therefore, we conducted a study based on ADR reports collected as part of the prospective active pharmacovigilance project on monitoring of ADRs in hospital wards (FORWARD). The primary aim of the present study was to determine the rate of ADRs occurring in hospitalized patients in departments of Internal Medicine. ADR-related hospital admissions, ADRs characteristics, preventability and their associated factors were also evaluated.

## Materials and Methods

### Setting

FORWARD (Facilitation of reporting in hospital ward) is an active pharmacovigilance project, funded by the Italian Medicines Agency (AIFA), carried out between January 2014 and December 2015 by a partnership of some hospital wards in the Sicilian region in Southern of Italy.

The study was performed in three wards of the Messina University hospital (Geriatrics, Internal Medicine and Metabolic diseases) and in three Internal Medicine wards of the Giarre, Acireale, and Caltagirone hospitals.

A monitor, specialist in clinical pharmacy, was assigned for each hospital ward. The monitors, who received specific training on pharmacovigilance, supported clinicians on the identifying of ADRs, through an accurate and systematic review of patients’ records.

The study was approved by the Ethics Committee of Messina University Hospital. All the patients provided written informed consent after a full explanation of the protocol design, and the study was conducted according to the Declaration of Helsinki.

### Data Collection

All patients admitted to participating hospitals during the 2 years were included in the study and followed until discharge. Patients were excluded if they were discharged within 24 h and/or had been transferred from other hospitals or other wards within the study hospitals. Data collected included sociodemographic characteristics, previous medical history, admission and discharge diagnoses, length of stay (LOS), laboratory tests, instrumental procedures, therapies administered (before admission and during hospital stay), medications prescribed at discharge, as well as information on the dosage, frequency, route, and indication of use of drugs. Data collected were entered into a computerized database developed *ad hoc*. Based on the collected data, all patients were classified into three different groups depending on whether they have developed at least one ADR (patients with ADR occurring during hospitalization and patients with ADRs that caused hospitalization) or not (patients without ADR).

All identified cases of ADRs were reviewed by a research team consisting of clinical pharmacologists, working at the Regional Pharmacovigilance Centre sited at University Hospital of Messina, ward physicians and monitors. The team analyzed each case of suspected ADR, to make a final causality assessment between a drug and an adverse reaction applying the Naranjo algorithm (Naranjo et al., 1981). Only ADR reports with a certain, probable, or possible causality assessment were included. In accordance with the Italian healthcare system, all collected ADRs were reported to the Italian Pharmacovigilance System. Additionally, for each ADR, a customized information-training feedback was elaborated, to update the reporters about risks related to a drug’s use.

Adverse drug reactions were codified as detailed by the Medical Dictionary for regulatory Activities (MedDRA^®^) (Brown et al., 1999) and organized according to the system organ class (SOC) classification and preferred term (PT). An ADR was considered serious when it was fatal, life-threatening, required or prolonged hospitalization, caused serious or permanent disability, or congenital anomaly/birth defect (European-Medicines-Agency, 2014). The preventability of ADRs was assessed according to Schumock and Thornton criteria (Schumock and Thornton, 1992).

The Anatomical Therapeutic and Chemical (ATC) classification was used to code therapeutic groups (level I–IV) and active principles (level V).

The diagnosis of admission and discharge at the hospital ward and concurrent diseases were coded according to the International Classification of Diseases, ninth edition, Clinical Modification (ICD-9-CM). Comorbidities were assessed by the Charlson score (Charlson et al., 1987; Quan et al., 2005).

The LOS was evaluated as the number of days between the date of admission and the date of discharge.

To determine the rate of ADRs occurring during hospital stay, the number of inpatients who experienced an ADR divided by the total number of patients admitted to the hospital wards was considered. The prevalence of ADRs present upon admission was calculated as the ratio between the number of patients admitted for ADRs and the total number of admissions in Internal Medicine departments.

### Statistical Analysis

For each of three above defined groups, descriptive statistical analyses were performed to evaluate basal demographic, clinical characteristics, LOS and drug-related variables of patients with and without ADRs. In the subjects with ADRs, suspected drugs, seriousness, outcome, preventability and type of ADR were also evaluated.

All results were expressed as medians with interquartile range (Q1–Q3) for continuous variables, and absolute and percentage frequencies for categorical variables. The Kolmogorov–Smirnov test for normality was performed to evaluate normal distribution. Since some of the numerical variables were not normally distributed, a non-parametric approach was used. We compared separately characteristics of patients with ADRs occurred during hospital stay vs. patients without ADRs and characteristics of patients admitted because of ADRs vs. patients without ADRs. In particular, the *U* Mann–Whitney test for independent sample was applied for continuous variables and two-tailed Pearson chi-squared test or Fisher test for categorical variables.

For each group, to identify predictors of ADRs, a univariate logistic regression model using hospitalized patients without ADRs as comparators was used to assess the possible influence of age, gender, number of drugs taken, and Charlson comorbidities index score. Moreover, all predictors were included in a stepwise multivariate logistic regression model (backward procedure, α = 5%). Odds ratios (ORs) with 95% confidence intervals (CIs) were calculated for each covariate of interest in univariate models (crude OR) and in multivariate model (adjusted OR). The goodness of fit of the regression model was assessed by the Hosmer–Lemeshow test for adequacy.

A *p*-value less than 0.05 was considered statistically significant. Statistical analysis was performed with SPSS version 20.0 (IBM Corp., SPSS Statistics).

## Results

### Study Population

During the study period, 4,802 patients were admitted to the participating wards. ADRs occurred in 153 patients (3.2%; 95% CI 2.7–3.7) during hospital stay. In 296 patients, ADRs were the cause of hospitalization, resulting in an estimated prevalence of ADR-related hospital admission of 6.2% (95% CI 5.5–6.8). In 17 patients ADRs occurred both before and during hospital stay. The main characteristics of patients are detailed in **Table 1**.

**Table 1:** Baseline characteristics of patients admitted to hospital wards.

	Patients without ADRs No. 4,370 (%)	Patients with ADRs during hospital stay No. 153 (%)	*p*-value^1^	Patients hospitalized due to ADRs No. 296 (%)	*p*-value^2^
**Sex**					
Females	2,189 (50.1)	89 (58.2)	0.049	188 (63.5)	<0.001
Males	2,181 (49.9)	64 (41.8)		108 (36.5)	
**F/M ratio**	1.0	1.4		1.7	
**Age median (Q1–Q3)**	78.0 (65.0–84.0)	77.0 (64.0–84.0)	0.495	78.0 (68.0–85.0)	0.169
**Age group**					
<65	1,074 (24.6)	39 (25.5)		60 (20.3)	
65–78	1,228 (28.1)	47 (30.7)		89 (30.1)	
79–84	1,010 (23.1)	31 (20.3)		71 (24.0)	
>84	1,058 (24.2)	36 (23.5)		76 (25.7)	
**Most frequent comorbidities**					
Diabetes mellitus	1,122 (25.7)	43 (28.1)	0.499	91 (30.7)	0.054
Hypertension	964 (22.1)	48 (31.4)	0.007	90 (30.4)	<0.001
COPD	796 (18.2)	30 (19.6)	0.661	39 (13.2)	0.029
Congestive cardiac failure	653 (14.9)	19 (12.4)	0.388	37 (12.5)	0.252
Ischemic heart disease	336 (7.7)	11 (7.2)	0.820	33 (11.1)	0.033
Arrhythmia	364 (8.3)	15 (9.8)	0.518	40 (13.5)	0.002
Renal diseases	622 (14.2)	22 (14.4)	0.060	66 (22.3)	<0.001
Liver diseases	375 (8.6)	12 (7.8)	0.748	17 (5.7)	0.088
Dementia	191 (4.4)	2 (1.3)	0.065	15 (5.1)	0.572
**Charlson score,** **median (Q1–Q3)**	1.0 (0.0–2.0)	1.0 (0.0–2.0)	0.856	1.0 (0.0–2.0)	0.105
**No. drugs taken at admission, median (Q1–Q3)**	4.0 (1.0–7.0)	5.0 (1.5–9.0)	0.002	6.0 (3.2–9.0)	<0.001
**No. drugs taken at admission**					
<4	2,325 (53.2)	67 (43.8)		103 (34.8)	
5–9	1,691 (38.7)	60 (39.2)		139 (47.0)	
≥10	354 (8.1)	26 (17.0)		54 (18.2)	
**LOS (days), median (Q1–Q3)**	9.0 (6.0-13.0)	12.0 (8.0–17.0)	<0.001	9.0 (6.0–12.0)	0.113


*ADRs, adverse drug reactions; LOS, length of stay; COPD, chronic obstructive pulmonary disease. ^*1*^Patients with ADRs during hospital stay vs. patients without ADRs. ^*2*^Patients hospitalized due to ADRs vs. patients without ADRs.*

In patients affected by ADRs during hospital stay, there was no significant difference in age (*p* = 0.495), and Charlson score (*p* = 0.856) respect to patients without ADRs. Conversely, most of them were female (*p* = 0.049), took more drugs daily (*p* = 0.002) and had a significantly longer hospital stay (*p* < 0.001) than the other group. Moreover, patients affected by ADRs during hospital stay were more likely hypertensive (*p* = 0.007).

In patients admitted for ADRs, median age (*p* = 0.169), Charlson comorbidities score (*p* = 0.105) and LOS (*p* = 0.113) was similar to patients without ADRs. However, females (*p* < 0.001) and patients in polytherapy (*p* < 0.001) were significantly more represented in this group of patients. Moreover, patients admitted because of ADRs were more likely affected by hypertension (*p* < 0.001), ischemic heart diseases (*p* = 0.033), arrhythmia (*p* = 0.002), renal diseases (*p* < 0.001), and less by chronic obstructive pulmonary diseases (*p* = 0.029) than patients without ADRs.

The number of drugs taken (OR 1.46, 95% CI 1.06–2.03; *p* = 0.022) and female gender (OR 1.39, 95% CI 1.03–1.93; *p* = 0.048) resulted independent predictors of ADR occurrence during the hospital stay when estimated both in univariate and multivariate approach. Conversely, age and comorbidities Charlson score did not affect the occurrence of ADRs (**Table 2A**).

**Table 2A:** Factors associated with adverse drug reaction occurred during hospital stay.

	OR_crude_ (95% CI)	*p*-value	OR_adjusted_ (95% CI)	*p*-value
**Female versus male**	1.39 (0.99–1.92)	0.050	1.39 (1.03–1.93)	0.048
**Age ≥ 85 years**	0.96 (0.67–1.41)	0.847	0.89 (0.61–1.31)	0.563
**No. drugs taken at admission ≥ 4**	1.46 (1.05–2.02)	0.023	1.46 (1.06–2.03)	0.022
**Charlson score ≥ 2**	0.98 (0.70–1.36)	0.891	0.91 (0.64–1.27)	0.571


*Hosmer–Lemeshow adequacy test, *p* = 0.966.*

Female gender (OR 1.75, 95% CI 1.37–2.24; *p* < 0.001) and the number of drugs (OR 2.14, 95% CI 1.67–2.74; *p* < 0.001), were also the only independent predictors of admission because of ADRs (**Table 2B**). Both the multivariate model adopted resulted adequate.

**Table 2B:** Factors associated with adverse drug reaction cause of hospitalization.

	OR_crude_ (95% CI)	*p*-value	OR_adjusted_ (95% CI)	*p*-value
**Female versus male**	1.73 (1.36–2.21)	<0.001	1.75 (1.37–2.24)	<0.001
**Age ≥ 85 years**	1.08 (0.83–1.42)	0.570	0.95 (0.72–1.25)	0.713
**No. drugs taken at admission ≥ 4**	2.13 (1.66–2.73)	<0.001	2.14 (1.67–2.74)	<0.001
**Charlson score ≥ 2**	1.12 (0.88–1.43)	0.348	0.97 (0.75–1.24)	0.782


*Hosmer–Lemeshow adequacy test, *p* = 0.937.*

### Characteristics of ADRs

A total of 153 inpatients developed 157 ADRs during their hospital stay, as some patients suffered more than one reaction. In 296 hospitalized patients for iatrogenic disease, 310 suspected adverse reactions were recorded.

#### In-Hospital ADRs

The 157 ADR reports were associated with 185 drugs which included 207 events (1.3 ADRs per report). Of the total ADRs, 44.6% were recognized as serious. A pharmacological interaction was suspected in four cases. In terms of outcomes, almost all patients experiencing ADRs completely recovered/improved (94.9%), one recovered with sequelae, two patients had not yet recovered, and in five cases the information was not available at the time of the report submission.

**FIGURE 1:**
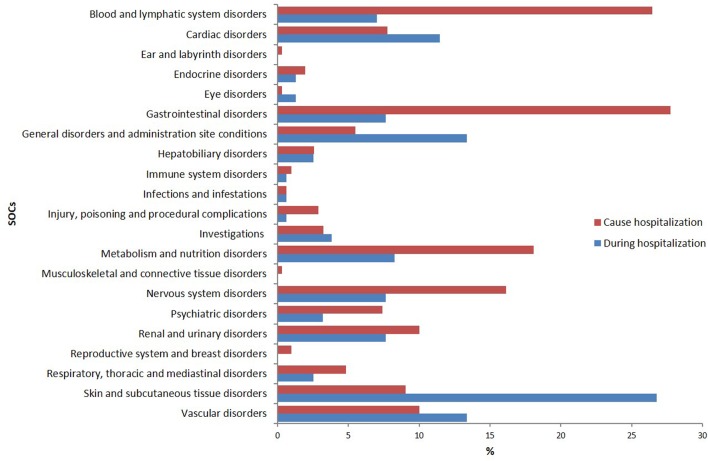
Distribution of reports of adverse drug reaction (%) by system organ class^a^ (SOC) according to MedDRA.^®^

According to MedDRA^®^ SOC classification, the most frequently reported ADRs were “cutaneous” (26.8%), “general” (13.4%), “vascular” (13.4%), and “cardiac” (11.5%) disorders (**Figure 1**).

**FIGURE 2:**
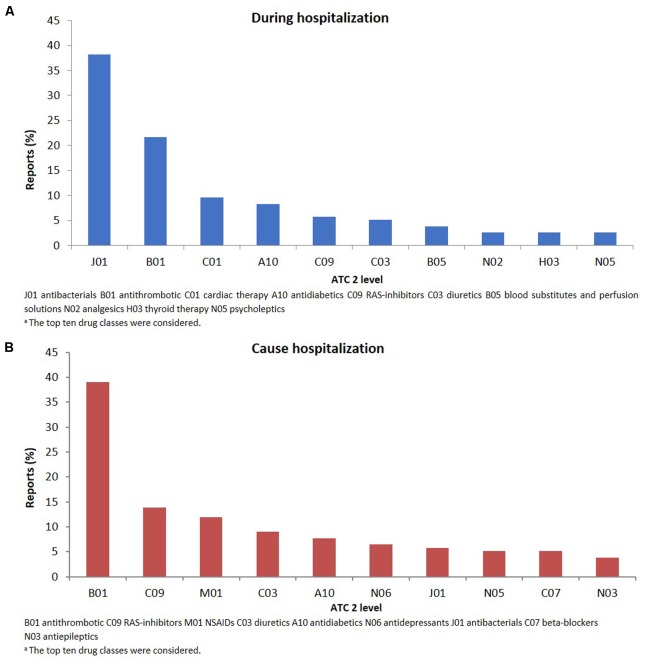
Drug classes^a^ (ATC second level) suspected to be responsible for the reported adverse drug reactions occurring during hospital stay **(A)** or leading to hospitalization **(B)**.

The therapeutic groups most frequently associated to ADRs were anti-bacterials (ATC J01: *N* = 60; 38.2%) and antithrombotic agents (ATC B01: *N* = 34; 21.7%), including heparins and platelet antiaggregants. Among anti-bacterials, quinolones (18.5%), and penicillins (7.0%) were the most frequent involved drugs. Other drug types associated with ADRs were cardiac therapy (ATC C01: *N* = 15; 9.6%), antidiabetics (ATC A10: *N* = 13; 8.3%), renin-angiotensin system (RAS) inhibitors (ATC C09: *N* = 9, 5.7%), diuretics (ATC C03: *N* = 8, 5.1%) and blood substitutes and perfusion solutions (ATC B05: *N* = 6, 3.1%) (**Figure 2A**). Anti-bacterials caused skin reactions (e.g., erythema, pruritus, and urticaria) and injection site reaction (**Table 3A**). Considering individual drugs, levofloxacin (*N* = 24; 15.3%) was the most common drug responsible for adverse reactions, followed by enoxaparin (*N* = 10; 6.4%), ivabradine (*N* = 6; 3.8%), piperacillin/beta-lactamase inhibitor (*N* = 6; 3.8%), and fondaparinux (*N* = 6; 3.8%). The main actions taken against detected ADRs were drug withdrawal (87.6%). Moreover, about 75% of drugs were originators.

**Table 3:** The most common drug-related events occurring during hospital stay **(A)** or leading to hospitalization **(B)**.

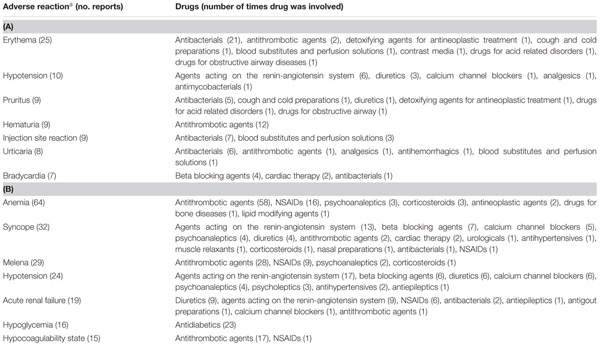

*^*a*^ADRs are classified by preferred terms according to MedDRA terminology. The sum of ADRs is higher than the total number of reports, since a single report could contain multiple ADRs.*

#### ADRs Leading to Hospital Admission

The 310 ADR reports were associated with 413 drugs which included 568 events (1.8 ADRs per report). ADR were related to drug overdose in 7 cases, in 8 to medication error, in 9 to drug–drug interactions, and in 10 to drug abuse. The leading cause for medication error were misunderstanding (*n* = 3), cognitive impairment (*n* = 2), and self-medication (*n* = 3). Almost 45% of ADRs that occurred during hospital stay were classified by physicians as serious. Complete recovery/improvement occurred in most of patients (87.7%), 17 (5.5%) had not yet recovered and 9 (2.9%) patients recovered with sequelae. In addition, three fatal cases (1.0 %) were reported during the follow-up. In nine cases (2.9%) the information was not available at the time of the report submission.

The SOCs most frequently associated with ADRs were “gastrointestinal” (27.7% of total ADR), “hematological” (26.5%), followed by “metabolic” (18.1%) and “nervous” disorders (16.1%) (**Figure 1**). Antithrombotic agents (ATC B01: *N* = 121; 39.0%) were the drug classes most commonly involved in hospital admissions, followed by RAS-inhibitors (ATC C09: *N* = 43; 13.9%), NSAIDs (ATC M01: *N* = 37; 11.9%), and diuretics (ATC C03: *N* = 28; 9.0%) (**Figure 2B**). The most common drug-event combinations were *anemia* or *melena* (20.6% and 9.4%) commonly associated with antithrombotics and NSAIDs, followed by *hypotension* or *syncope* (7.7% and 10.3%) associated with RAS-inhibitors and *acute renal failure* (6.1%), in which the most involved drug types were RAS-inhibitors with or without concomitant diuretics and/or NSAIDs (**Table 3B**). With regard to individual drugs, acetylsalicylic acid has been reported as suspected in 50 cases (16.1%), followed by acenocoumarol and furosemide in 29 and 21 cases, respectively. In the majority of cases of ADRs, the suspected drugs were suspended (89.3%), in 8.5% they were continued, and in 2.2% the dosage was reduced. Also, about 80% of them were originators.

### Preventability of ADRs

**FIGURE 3:**
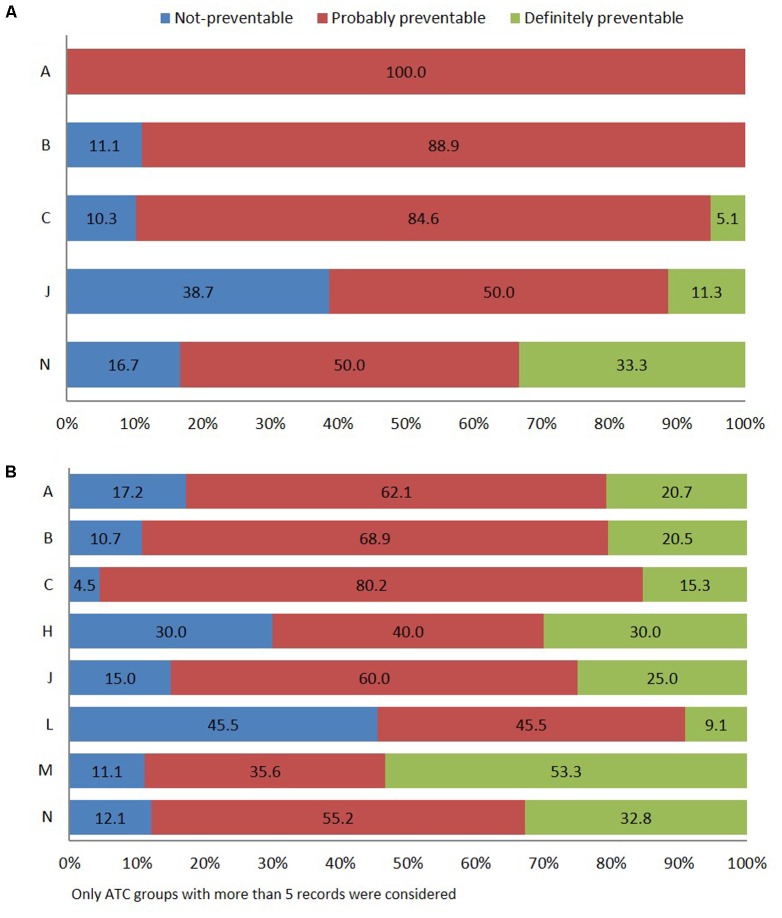
Distribution of ADR reports occurring during hospital stay **(A)** or leading to hospitalization **(B)** using Schumock and Thornton preventability scale.

According to Schumock and Thornton algorithm (Schumock and Thornton, 1992), 69.4% of in-hospital ADRs were related to probably preventable ADR, 24.2% to unavoidable, and 6.4% to certainly preventable. The most frequent types of drugs involved in probably preventable ADR belong to group “*A*” of the ATC classification (gastrointestinal tract and metabolism, 100.0%), specifically antidiabetics, followed by “*B*” (blood and hematopoietic organs, 88.9%), and “*C*” (cardiovascular system, 84.6%), mostly cardiac therapy (*N* = 13) and RAS-inhibitors (*N* = 9). Other pharmacological groups were antimicrobials for systemic use (50.0%) and nervous system (50.0%) (**Figure 3A**).

Adverse drug reaction-related admissions were mainly attributed to probably preventable ADR (63.9%). The drugs of the musculoskeletal system (*M*) (51.1%), specifically, the NSAIDs (M01A) were associated with “definitely avoidable” ADRs (**Figure 3B**). Finally, definitely preventable ADRs were significantly associated with ADR-cause of hospitalization (23.2%; 95% CI 18.5–27.9) respect to ADRs occurred during hospital stay (6.4%; 95% CI 2.5–10.2) (*p* < 0.001). Otherwise, a significantly higher number of not-preventable ADRs were associated with adverse reactions occurring during hospital stay (24.2%; 95% CI 17.5–30.9 vs. 12.9%; 95% CI 9.2–16.6) (*p* = 0.002).

## Discussion

It is widely acknowledged that older patients are mainly at risk for ADRs (Thomas and Brennan, 2000; Oteri et al., 2010; Steinman and Hanlon, 2010), primarily due to increased chronic disease, polypharmacy (concomitant prescription of five or more drugs), and age-related physiological changes affecting the pharmacokinetics and pharmacodynamics of drugs (Martin et al., 1998; Lagnaoui et al., 2000; McDonnell and Jacobs, 2002; Zopf et al., 2009; Ruiter et al., 2012). van der Hooft et al. (2006) found that older people have more than 10-fold prevalence rate (9.8%) of ADRs compared to younger populations (0.4%). Internal Medicine departments represent a useful setting for studying the incidence of ADRs in older patients. In fact, hospitalized patients have several comorbidities, take multiple drugs at hospitalization and frequently suffered from renal failure (Ingrasciotta et al., 2014, 2015; Trifiro et al., 2014). Our study provides useful information on the clinical impact of ADRs through a survey of several hospital wards that included a substantial number of patients, over a 2-year period.

In our study, the in-hospital incidence rate of ADRs was 3.2/100 patients admitted and it was consistent with findings of several previous studies. Indeed, different studies report a range from 1.7 to 50.9% (Bordet et al., 2001; Brennan et al., 2004; Aranaz-Andres et al., 2008; Dequito et al., 2011; Dupouy et al., 2013), depending on differences in data collection methods, definitions of ADRs, studied population, settings in which the studies were performed. The onset of at least one ADR during hospitalization was associated with a 4 days’ stay median prolongation, similarly to be observed by Nobili et al. (2011) in a cohort of 1,332 elderly patients hospitalized in 38 different Italian hospitals.

The drug-related adverse reactions are also an important cause of hospital admission. The rate of ADR-related hospitalization found in this study (6.2%) was similar to those found by other authors (Lazarou et al., 1998; Howard et al., 2003; Pirmohamed et al., 2004; Franceschi et al., 2008; Kongkaew et al., 2008; Leendertse et al., 2008). A recent systematic review of 21 studies reported that the median prevalence rate of hospitalization was 7.0% (Al Hamid et al., 2014). Another review, focused on European studies, reported that the percentage of hospitalizations caused by ADRs (3.6%) was lower than those reported in earlier reviews. In addition, the authors found that up to 10.0% of patients in European hospitals experience an ADR during their stay (Bouvy et al., 2015).

Using the multivariate models, female gender and number of drugs assumed were the only predictors associated both with the occurrence of ADRs during hospital stay or with the admission because of ADR. These findings are consistent with those observed in other studies (Fauchais et al., 2006; Nguyen et al., 2006; Davies et al., 2007; Madeira et al., 2007; Zopf et al., 2008a,b, 2009). It is also conceivable that the different susceptibility to ADRs between males and females is due to physiological characteristics, such as weight, intestinal transit velocity and fat percentage, as well as genetic/metabolic and hormonal differences (Soldin et al., 2011). On the contrary, unlike previous studies (Pirmohamed et al., 2004), the average age of patients is similar in patients with and without ADRs and the absence of association was found also in the multivariate models. This, could be explained by our focus on Internal Medicine wards that included patients with a high average age. Indeed, in similar setting based on elderly patients (Dupouy et al., 2013), age was not found a predictive factor of ADRs occurrence.

It is known that polypharmacy is strongly associated with comorbidity, both of which lead to a higher risk of ADR (Nguyen et al., 2006; Davies et al., 2007). The Charlson comorbidity index (Charlson et al., 1987), weighting comorbid conditions (comorbidities), has been widely utilized by health researchers to measure burden of disease. Comorbidities, as well as the number of drugs assumed, were associated with the occurrence of adverse drugs events in several studies (Madeira et al., 2007; Davies et al., 2009; Mohebbi et al., 2010). However, in different setting, like post emergency unit, less adverse drug events occurred in inpatients with comorbid conditions (Dupouy et al., 2013). In our study, Charlson comorbidity index was not associated with the occurrence of ADRs in the multivariate models. The different findings obtained by different studies could be explained by the variability of the range of comorbidities considered in Charlson index. In our study, some comorbid conditions such as diabetes, renal and liver diseases were frequently reported, due to specialized branches in these healthcare settings, while dementia or neoplasms were less frequent because of specific oncology and neurology/psychiatric departments in the hospitals involved. Moreover, the effect of comorbidities on ADRs occurrence could be partially avoid by the precautions taken by doctors or care givers with frail patients. Conversely, according to literature, our study confirmed that the number of drugs assumed is independently associated with the occurrence of ADRs during hospital stay, as well as in patients admitted because of ADRs.

Drugs involved in hospital drug-related reactions were primarily anti-bacterials and antithrombotic agents. This is quite consistent with data found in a prospective study carried out in Italy (Conforti et al., 2012). On the other hand, in the study by Davies et al. (2009), electrolyte disturbances associated with diuretics were the most frequently occurring ADR. This difference is probably due to higher rate of parenteral antibiotics prescribed in Italy compared to the Northern European countries (Davies et al., 2009). Moderate to severe skin/allergic reactions (Polimeni et al., 2016) and application-site disorders represented the most common ADRs observed. These results might be partially explained by the higher rate of parenteral antibiotics among inpatients and the involvement of nurses and physicians in the project with a higher sensitiveness to identify ADRs.

Drugs causing hospitalization were similar with those found in previous studies, where antithrombotic, RAS-inhibitors, NSAIDs and diuretics were most frequently associated with ADRs. These drug groups, commonly used in elderly patients, have a high innate toxicity and require close monitoring for their safe use. Clinically significant adverse reactions such as anemia/melena, syncope/hypoglycemia and acute renal failure were the most frequently events, as previously observed (Mjorndal et al., 2002; Onder et al., 2002; Pirmohamed et al., 2004; Howard et al., 2007; Franceschi et al., 2008; Leendertse et al., 2008; van der Hooft et al., 2008; Hofer-Dueckelmann et al., 2011; Ruiter et al., 2012).

A key aspect in the study of ADRs is the possibility of prevention. More than half of ADRs identified in our study were preventable and, as a consequence, avoidable. This finding is in agreement with data from studies performed in the United Kingdom (63.0%) and United States (67.0%) (Howard et al., 2003; Pirmohamed et al., 2004). Two meta-analyses of observational studies showed a proportion of preventable ADR-related hospital admissions ranging between 28.9% and 52.0% (Beijer and de Blaey, 2002; Hakkarainen et al., 2012). Another study reports that 19.4% of hospitalizations were caused by ADRs, and 65% of them were preventable (Perez Menendez-Conde et al., 2011). Moreover, drug classes mainly involved in preventable ADRs were quite consistent with the systematic review of Howard et al. (2007) who found that antiplatelet drugs, diuretics, NSAIDs and anticoagulants are the most involved drugs in preventable ADRs. (Mjorndal et al., 2002; Onder et al., 2002; Pirmohamed et al., 2004; Howard et al., 2007; Franceschi et al., 2008; Leendertse et al., 2008; van der Hooft et al., 2008; Hofer-Dueckelmann et al., 2011; Ruiter et al., 2012).

Despite the large number of studies conducted to estimate the true incidence of preventable ADRs, further prevention strategies are needed to improve the safety of prescribing and monitoring drug and improve adherence to medication.

### Limits and Strengths

This study will provide detailed information on the prevalence and characterization of ADRs among inpatients. It is a multicenter study to prospectively identifies the cases of ADR for a long period. The prospective model allowed very accurate data collection of patient drug history and causality/preventability assessments. Moreover, the identification and assessment of ADRs was made by trained professionals, through computerized monitoring programs. Finally, this study provides updated information on ADR related hospital admissions that may allow the design of preventive strategies. However, since our study was conducted in Internal Medicine wards, the generalization of our results to other settings is not possible. Moreover, some comorbid condition such as diabetes, renal and liver diseases were frequently reported due to specialized branches in these healthcare settings, while others, like neoplasms or mental disorders, are less frequent due to specific oncology and neurology/psychiatric departments in the hospitals involved. Only a very small proportion of treatment harms are generally reported in hospital records. As a consequence, we cannot exclude the possibility that some drug-related events were not recorded because of the lack of clinical monitors during weekends. Finally, the exclusion of patients discharged within 24 h could lead to underestimation of ADR-related hospitalization. However, we preferred to not analyze these medical records due to poor quality and availability of date on drug-related harms.

Our purpose did not include evaluation of the intervention of clinical pharmacologists on reducing the number of adverse reactions in-hospital setting. However, our findings highlight the importance of the “monitors,” dedicated to pharmacovigilance, to improve a correct quality reporting. Furthermore, drug-event reviews led by expertise presence has been considered useful and well accepted by physicians.

## Conclusion

These results indicate that ADRs that occur during hospitalization or contributing to admission to Internal Medicine wards are considerable, and gender and polypharmacy are associated with their occurrence. The high incidence of preventable ADRs provides a strong rationale for undertaking future research aimed to implement interventions useful to reduce drug-related reactions.

## Author Contributions

All authors listed have made substantial, direct contribution to the work, and approved it for publication. VA: project coordination. CG, GR, GB, GM, FR, and EM: acquisition of data. RF: informatics support. CG, VA, and PC: analysis and interpretation of data. CG and VA: drafting of manuscript. ES and VA: critical revision.

## Conflict of Interest Statement

The authors declare that the research was conducted in the absence of any commercial or financial relationships that could be construed as a potential conflict of interest.
